# Myc and Miz-1 have coordinate genomic functions including targeting *Hox *genes in human embryonic stem cells

**DOI:** 10.1186/1756-8935-4-20

**Published:** 2011-11-04

**Authors:** Natalia Varlakhanova, Rebecca Cotterman, Keith Bradnam, Ian Korf, Paul S Knoepfler

**Affiliations:** 1 Department of Cell Biology and Human Anatomy, University of California Davis School of Medicine, Sacramento, CA, USA; 2 Genome Center, University of California Davis School of Medicine, Sacramento, CA, USA; 3 Institute of Pediatric Regenerative Medicine, Shriners Hospital For Children Northern California, Sacramento, CA, USA; 4 Department of Molecular and Cell Biology, University of California Davis School of Medicine, Sacramento, CA, USA

## Abstract

**Background:**

A proposed role for Myc in maintaining mouse embryonic stem (ES) cell pluripotency is transcriptional repression of key differentiation-promoting genes, but detail of the mechanism has remained an important open topic.

**Results:**

To test the hypothesis that the zinc finger protein Miz-1 plays a central role, in the present work we conducted chromatin immunoprecipitation/microarray (ChIP-chip) analysis of Myc and Miz-1 in human ES cells, finding homeobox (*Hox*) genes as the most significant functional class of Miz-1 direct targets. Miz-1 differentiation-associated target genes specifically lack acetylated lysine 9 and trimethylated lysine 4 of histone H3 (AcH3K9 and H3K4me3) 9 histone marks, consistent with a repressed transcriptional state. Almost 30% of Miz-1 targets are also bound by Myc and these cobound genes are mostly factors that promote differentiation including *Hox *genes. Knockdown of Myc increased expression of differentiation genes directly bound by Myc and Miz-1, while a subset of the same genes is downregulated by Miz-1 loss-of-function. Myc and Miz-1 proteins interact with each other and associate with several corepressor factors in ES cells, suggesting a mechanism of repression of differentiation genes.

**Conclusions:**

Taken together our data indicate that Miz-1 and Myc maintain human ES cell pluripotency by coordinately suppressing differentiation genes, particularly *Hox *genes. These data also support a new model of how Myc and Miz-1 function on chromatin.

## Background

Miz-1 is a member of the POZ domain/zinc finger transcription factor family. It contains 13 zinc fingers and a POZ/BTB (BTB for BR-C, ttk and bab, POZ for Pox virus and zinc finger) domain at its N-terminus [1]. In cancer cell lines, Miz-1 binds to specific sequences termed initiator elements (INR) in the core promoters of its target genes and activates their transcription through recruitment of coactivators including the histone acetyltransferase (HAT) p300 and nucleophosmin [2-4]. Among previously identified Miz-1 regulated targets are negative regulators of cell cycle control and cell growth, including *p15Ink4b*, *p21Cip1*, and *c/EBPα *[3,5,6]. By activating negative regulators of cell cycle, Miz-1 has a growth arrest function. In addition, Miz-1 interacts with Myc and recruits it to the promoters of its target genes to repress transcription [1]. Miz-1 also functions with repressor proteins such as Bcl-6, Zbtb4, and Gfi-1 [7-9]. Upon binding to Myc, transcriptional activation by Miz-1 is inhibited and Myc/Miz-1 complexes act as transcriptional repressors. Myc represses transactivation by Miz-1 at least in part by competing with p300 and nucleophosmin for binding to Miz-1 [3,4]. The Myc/Miz-1 complex also recruits the DNA methyltransferase, DNA (cytosine-5)-methyltransferase 3A (Dnmt3a), and histone deacetylases (HDACs) to gene promoters leading to silencing of gene expression [10,11]. Myc, therefore, overcomes Miz-1-induced growth arrest by binding to Miz-1 to repress target genes involved in cell cycle regulation.

*Myc *genes were initially characterized as proto-oncogenes and the proteins they encode belong to the family of basic helix-loop-helix zipper transcription factors [12]. Myc proteins regulate normal proliferation, cell growth, and apoptosis, cellular functions aberrantly regulated by excess Myc during malignant transformation (reviewed in [13]). Myc has the ability to both positively and negatively regulate transcription. The most thoroughly studied and understood function of Myc is its ability to activate genes via binding to specific DNA sequences called E-boxes with its partner protein, Max [14-16]. The Myc/Max complex recruits several coactivators and HATs to DNA such as transformation/transcription domain-associated protein (TRRAP), Gcn5 and Tip60, which leads to promoter activation [17,18]. c-Myc also binds to positive transcription elongation factor b (p-TEFb) and contributes to pause release in embryonic stem (ES) cells thus promoting transcription from its target genes [19,20]. Myc represses transcription at least in part by targeting Miz-1 with important biological consequences. For example, Myc regulates keratinocyte differentiation [21] and enhances self-renewal of neural progenitor cells (NPCs) [22] via binding to Miz-1. The Myc/Miz-1 complex in addition inhibits the differentiation of preadipocytes to adipocytes in culture, a process which is controlled by the transcription factor CCAAT/enhancer binding protein alpha (c/EBP-a) [23]. Furthermore, the Myc/Miz-1 complex suppresses the expression of *Mad4*, which plays an important role in the control of cellular proliferation and differentiation in mouse erythroleukemia (MEL) cells [24].

Myc plays critical roles in maintenance of mouse ES cell pluripotency and self-renewal, as well as induction of pluripotency during induced pluripotent stem (iPS) cell formation [25-29]. Myc maintains and induces pluripotency at least in part by repressing differentiation-associated gene expression, but the molecular mechanisms of Myc repression of differentiation genes in stem cells are yet to be fully defined. Much the same as Myc, Miz-1 knockout embryos are not viable, suggesting important, but as yet uncharacterized roles for Miz-1 in stem cells and early embryogenesis [30]. No unbiased, global genomic studies have been reported for Miz-1 leaving its transcriptional function less clear than that of Myc. We hypothesized that Myc and Miz-1 function coordinately together in ES cells to delineate the expression of differentiation-associated genes. To test this hypothesis we conducted functional genomics studies for Miz-1, focusing on human ES cells and in parallel characterizing Myc genomic function in the same cells. Our findings define an important new role for Myc and Miz-1 in regulating differentiation-related gene expression, particularly homeobox (*Hox*) gene expression, in human ES cells.

## Results

### Analysis of genome-wide Miz-1 binding in human ES cells indicates *Hox *genes as the most common targets

**Figure 1:**
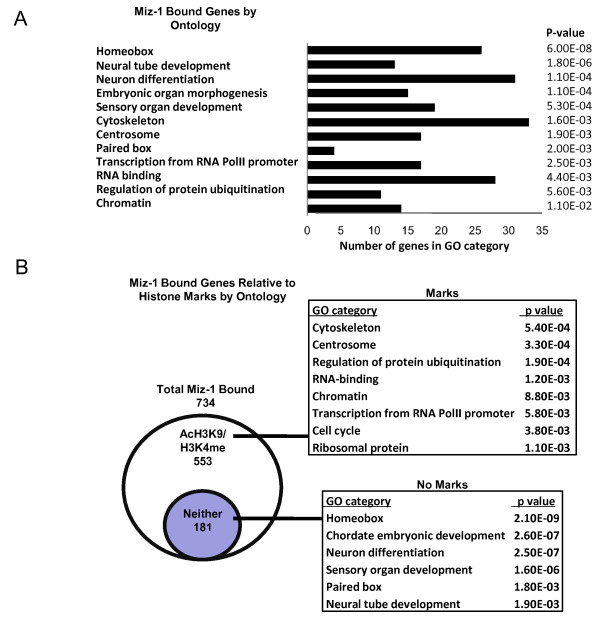
**Genomic binding of Miz-1 and functional ontology of its target genes in human embryonic stem (ES) cells. (A) **Gene ontology (GO) functional annotation of Miz-1 bound genes was performed using DAVID functional annotation software. The most significant biological process GO terms are shown. The number of genes belonging to each category within the Miz-1 bound population is indicated by black bars. **(B) **Acetylated lysine 9 and trimethylated lysine 4 of histone H3 (AcH3K9 and H3K4me3) status for Miz-1 bound target genes. *P *values are indicated in all cases.

We conducted chromatin immunoprecipitation/microarray (ChIP-chip) analysis to define Miz-1 genomic DNA binding in human ES cells. For the ChIP-chip analysis we used the 2.1 M Deluxe promoter array platform (Roche Nimblegen, Indianapolis, IN) and a well characterized Miz-1-specific antibody [31] from biological replicates of exponentially growing human ES cells. We identified 734 genes bound by Miz-1 in both replicate samples. Regions of Miz-1 binding were functionally annotated for gene ontology (GO; http://david.abcc.ncifcrf.gov/), indicating that Miz-1-occupied genes are involved in embryonic development, differentiation, chromatin packaging, as well as RNA binding and protein ubiquitination processes (Figure 1A). Importantly, the most significant class of Miz-1 direct target genes was *Hox *genes (*P *value = 6E-8) including *HOXB2*, *HOXB6*, *VAX2*, *MEIS1*, *PAX3*, *PAX7 *and others. *Nanog *and *POU5F1 *were also among Miz-1 target genes [see Table S1 in Additional file 1 for a comprehensive list of Miz-1 target genes].

### A dichotomous relationship of Miz-1 genomic binding to histone marks associated with transcriptional activation

To determine if there is an association between Miz-1 binding and actively transcribed euchromatic domains, in parallel to Miz-1 ChIP-chip, we performed ChIP-chip analysis in ES cells using antibodies specific to acetylated lysine 9 and trimethylated lysine 4 of histone H3 (AcH3K9 and H3K4me3), which are known marks of transcriptionally active, euchromatic domains. We compared Miz-1 bound target genes to regions of H3K4me3 or AcH3K9 enrichment (Figure 1B; [see Table S1 in Additional file 1]). Transcriptional activation marks H3K4me3 and AcH3K9 were found on 553 Miz-1 target genes, which included genes primarily involved in RNA binding, cytoskeleton, protein ubiquitination, cell cycle and regulation of transcription. *Nanog *and *POU5F1*, encoding known pluripotency factors, are Miz-1 bound and also enriched in AcH3K9 and H3K4me3, consistent with their active gene expression state in human ES cells. Importantly, specific individual peaks of H3K4me3 and AcH3K9 overlapped with Miz-1 binding peaks, suggesting a tight association between Miz-1 and these marks. We also found 181 Miz-1 target genes that were not associated with H3K4me3 and AcH3K9 marks, including most prominently *Hox *genes, but also other genes involved in development and differentiation.

To determine which of the Miz-1 bound genes are enriched in the H3K27me3 silencing histone modification, we compared genes bound by Miz-1 with genes previously reported to contain significant H3K27me3 in human ES cells [32]. We found that out of the 181 Miz-1 bound genes that are not associated with H3K4me3 and AcH3K9, 45 genes contain H3K27me3 (25%). In contrast, out of 553 Miz-1 bound genes associated with activation marks only 36 genes have H3K27me3 (6%) [see Table S1 in Additional file 1]. Examples of such comodified Miz-1 bound genes include several *Hox *genes such as *MEIS2, PAX6, GSC*, and *SHOX2*.

### Identification of genes bound by c-Myc and N-Myc in human ES cells

**Figure 2:**
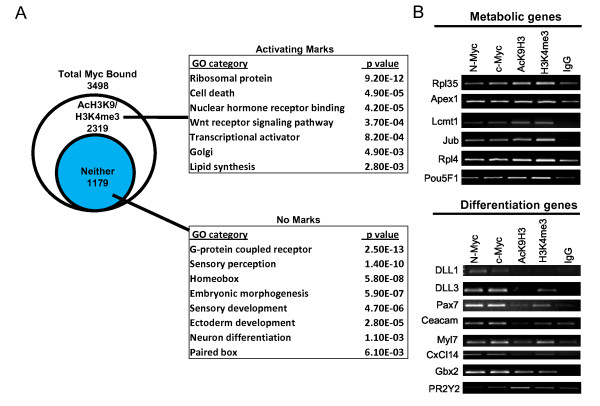
**Genomic binding of Myc and functional ontology of its target genes in human embryonic stem (ES) cells**. **(A) **Acetylated lysine 9 and trimethylated lysine 4 of histone H3 (AcH3K9 and H3K4me3) status for Myc bound genes. Gene ontology analysis for Myc bound genes with or without enrichment in histone activation marks H3K4me3 and AcH3K9 was performed using DAVID functional annotation software. *P *values are indicated. Myc binding events that do not overlap with either histone modification are represented by the blue circle. **(B) **Confirmation of Myc direct targets identified by chromatin immunoprecipitation/microarray (ChIP-chip). ChIP was performed using antibodies to c-Myc, N-Myc, AcH3K9, and H3K4me3. *PR2Y2 *gene, not bound by either c-Myc or N-Myc, was used as a negative control.

Because Miz-1 is known to bind to Myc, for comparison we analyzed Myc genomic binding in human ES cells, focusing on c-Myc and N-Myc, the predominant Myc proteins expressed in ES cells. Using ChIP-chip analysis, we identified 3,498 putative direct Myc bound target genes (c-Myc and/or N-Myc bound) (Figure 2A; [see Table S2 in Additional file 1]). By comparing Myc target genes with regions of H3K4me3 or AcH3K9, we found that most Myc-bound genes (2,319 genes; 66%) are associated with active histone modifications [see Table S2 in Additional file 1]. These putative targets of Myc transcriptional activation encode proteins involved in metabolism, ribosomal biology, apoptosis and signaling (Figure 2A). Notably, out of 3,498 Myc target genes, about one-third (1,179 genes) are not associated with H3K4me3 and/or AcH3K9 [see Table S2 in Additional file 1]. Many of the potential targets of Myc repression encode transcription factors involved in differentiation such as *PAX7*, *DLL1*, *DLL3*, and numerous *Hox *genes (Figure 2A). We further validated a subset of Myc binding targets identified in our ChIP-chip experiments and their associated histone mark profiles by employing direct ChIP assays with c-Myc, N-Myc, AcH3K9 and H3K4me3 antibodies (Figure 2B), with all those tested being validated. *PR2Y2*, which is not bound by either N-Myc or c-Myc, was used as a negative control for Myc binding. The relative abundance of AcH3K9 and H3K4me3, as suggested by the ChIP-chip assays, was strikingly different in direct ChIP assays at metabolic/pluripotency genes (high levels) versus differentiation-associated genes (nearly absent levels; Figure 2B).

### Miz-1 and Myc co-occupy the promoters of *Hox *genes and other differentiation-associated genes

**Figure 3:**
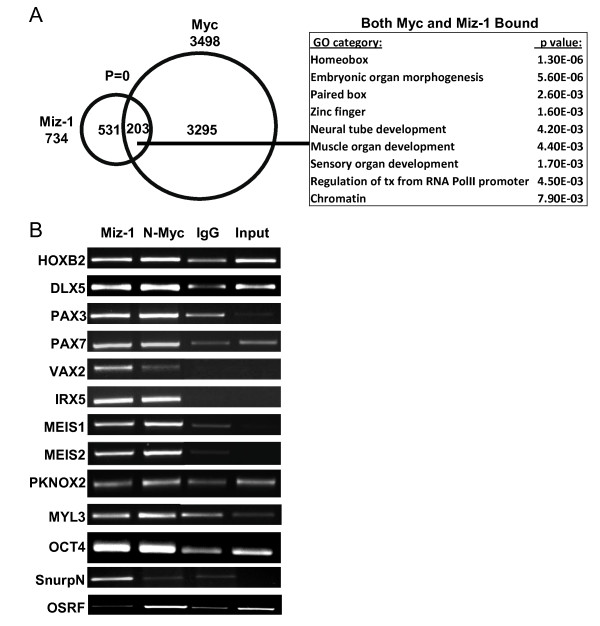
**Myc and Miz-1 are cobound on differentiation-associated genes**. **(A) **Venn diagram showing overlap of Miz-1 and Myc bound genes. The most significant biological process gene ontology (GO) terms are shown on the right with *P *values. **(B) **Chromatin immunoprecipitation (ChIP) confirmation of Miz-1 and Myc common target genes identified by ChIP/microarray (ChIP-chip) analysis. *SnurpN *and *OSRF *genes, identified to be bound respectively only by Miz-1 or only by N-Myc by ChIP-chip, were used as a negative control.

**Figure 4:**
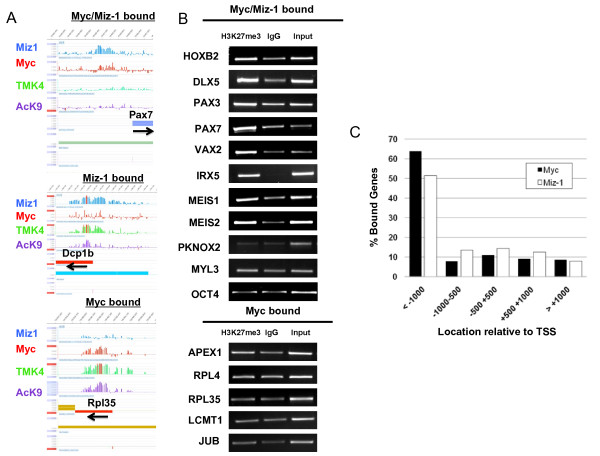
**Target genes bound by both Myc and Miz-1 have a distinct, transcriptionally inactive profile of histone marks**. **(A) **Examples of enrichment profiles observed for Myc, Miz-1, acetylated lysine 9 of histone H3 (AcH3K9) (AcK9) and trimethylated lysine 4 of histone H3 (H3K4me3) (TMK4) for Myc/Miz-1, Miz-1 alone or Myc alone bound genes are shown. Miz-1, Myc, TMK4, and AcK9 are indicated in blue, red, green, and purple, respectively. Black horizontal arrows indicate direction of transcription. Peaks are on a Log2 scale. Gene names are indicated above the colored horizontal bars with tiled regions immediately below and transcriptional start sites (TSSs) are indicated by short vertical lines near the bottom. **(B) **Chromatin immunoprecipitation (ChIP) analysis of H3K27me3 enrichment on Myc/Miz-1 and Myc alone bound genes. **(C) **Myc and Miz-1 binding distribution relative to the TSS.

To determine if Miz-1 and Myc bind and regulate common sets of genes, we compared Miz-1 target genes with the genomic targets of Myc. We found very significant overlap as 203 genes out of 734 Miz-1 target genes are co-occupied by Myc (*P *value = 0) (Figure 3A; [see Table S3 in Additional file 1]). Miz-1/Myc target genes included *Hox *genes and genes involved in neural and muscle differentiation including *HOXB2, PAX3, PAX7*, and *VAX2*. We further validated a subset of Myc/Miz1 binding targets by direct ChIP analysis with Miz-1 and N-Myc antibodies (Figure 3B), with all those tested being validated. *SnurpN or OSRF*, which are bound respectively only by Miz-1 or only by N-Myc based on our ChIP-chip data, were used as negative control for N-Myc and Miz-1 binding, respectively. Examples of ChIP-chip enrichment profiles observed for Myc, Miz-1, AcH3K9 and H3K4me3 for Myc and Miz1 target genes are presented in Figure 4A, showing actual peak data. The majority of differentiation-associated genes bound by both Myc and Miz-1 exhibit a repressive profile of low/absent AcH3K9 and H3K4me3. When compared to the H3K27me3 whole genome binding profile reported previously [32], 35% of Myc and Miz-1 cobound genes with low/absent AcH3K9 and H3K4me3 are enriched in H3K27me3, further demonstrating their repressed state. Myc/Miz-1 bound differentiation-associated target genes with low levels of AcH3K9 and H3K4me3 in our ChIP-chip assays also showed very high relative levels of H3K27me3 (Figure 4B). In contrast, Myc and Miz-1 cobound genes such as *OCT4, PKNOX2 *and *MYL3 *that also have robust peaks of AcH3K9 and H3K4me3 in their promoters, suggesting they are actively transcribed, do not have significant H3K27me3. The metabolic genes bound by N-Myc but not by Miz-1 are also not marked by H3K27me3, which fits with their transcriptionally active state (Figure 4B).

We analyzed the specific regions of Miz-1 and Myc genomic binding using location analysis (NimbleScan software, Roche Nimblegen, Indianapolis, IN), annotating them to the nearest transcriptional start site (TSS; Figure 4C). Interestingly, we found that the majority of genomic regions for both Miz-1 and Myc binding (all binding sites, not just cobound sites) are located at least 1 kb upstream of TSSs (Miz-1 is 51%, Myc is 63%), while the current model of Miz-1 binding, not based on functional genomics data, suggests that Miz-1 binding elements (INR) are predominantly localized in the core TSS region. The binding pattern for Myc that we found, however, is in agreement with previously reported location analysis of Myc binding sites in the human genome [33]. Supporting the notion of linked Myc and Miz-1 function, in more than half of Myc and Miz-1 cobound genes (109 out of 203 genes) actual binding loci of Myc and Miz-1 are in close proximity to one another (peaks are located within 250 bp of each other), suggesting that Myc and Miz-1 factors work through shared genomic domains, and function either within common protein complexes or within shared, small cis-regulatory elements [see Table S3 in Additional file 1].

To determine whether the experimentally identified Myc and Miz-1 cobound sites possess a consensus motif that might be utilized by the Myc/Miz-1 complex, we used MEME [34], but no consensus motif was found among Miz-1 or Myc/Miz-1 bound sequences. In contrast, Myc target loci show a substantial enrichment in Myc-binding consensus E-box motifs (CACGTG) (*P *value = 9.7E-277) when analyzed by Cis-regulatory Element Annotation System (CEAS; http://liulab.dfci.harvard.edu/CEAS/) [35]. However, no enrichment in E-box motifs in target loci simultaneously bound by Myc and Miz-1 was found, which indicates that the binding of the Myc/Miz-1 complex to its target genes is likely most often E-box-independent.

### Myc knockdown in human ES cells leads to growth arrest and upregulation of differentiation-associated target genes

**Figure 5:**
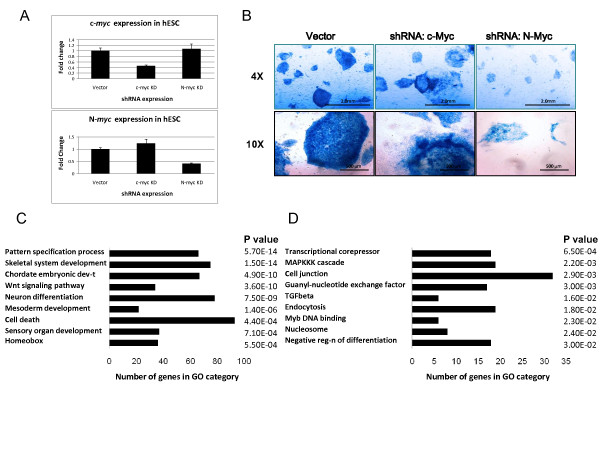
**Loss of Myc leads to spontaneous differentiation and upregulation of differentiation-associated genes**. **(A) **Confirmation of c-Myc or N-Myc knockdown by quantitative real-time (qRT)-PCR PCR. Levels of Myc RNAs measured by qRT-PCR were normalized to the levels of loading control peptidylprolyl isomerase A (PPIA; cyclophilin A). Error bars are standard deviations. N = 3. **(B) **Alkaline phosphatase staining of vector alone or Myc small hairpin (sh)RNA human embryonic stem (ES) cell colonies. Images were taken at either 4 × (top panels) or 10 × magnifications (bottom panels). **(C) **Gene ontology (GO) analysis of genes that are differentially expressed upon Myc knockdown with indicated *P *values.

We hypothesized that by disrupting Myc expression in human ES cells, repression by the Myc/Miz-1 complexes would be relieved leading in turn to the upregulation of differentiation-associated target gene expression. To test this idea and to evaluate the effect of Myc loss-of-function on global gene expression changes in human ES cells, we employed lentiviral vectors to introduce small hairpin (sh)RNAs designed to specifically knockdown (KD) either endogenous c-Myc or N-Myc. We observed approximately 60% decreases in c-Myc and N-Myc RNA levels, respectively, 5 days after separate introduction of each shRNA (Figure 5A). Myc protein levels were more substantially decreased (threefold to fourfold) upon introduction of the Myc shRNAs [see Figure S1 in Additional file 2]. Simultaneous KD of c-Myc and N-Myc completely inhibited the formation of viable colonies (not shown). In the context of both the independent c-Myc KD and the N-Myc KD, 5 days after shRNA introduction, human ES cells exhibited a significant inhibition of colony growth and size, accompanied by colony morphological changes characteristic of spontaneous differentiation (Figure 5B). Consistent with these changes, alkaline phosphatase (AP) staining, a marker of undifferentiated ES cells, decreased in c-Myc and N-Myc KD ES cells relative to control ES cells (Figure 5B).

**Figure 6:**
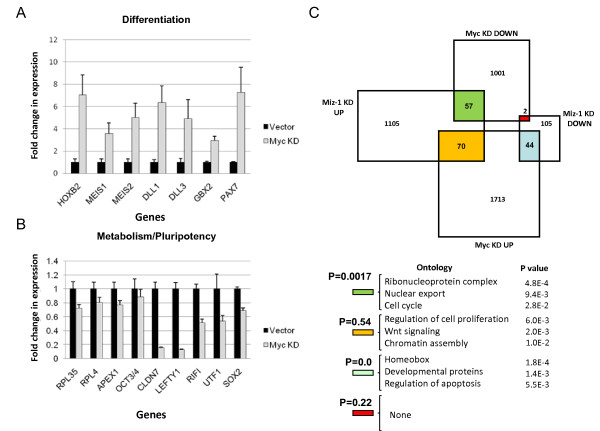
**Myc represses expression of differentiation-associated including homeobox (*Hox*) genes, a function antagonized by Miz-1**. **(A) **Confirmation of gene expression changes in c-myc knockdown (KD) embryonic stem (ES) cells, determined by gene expression array, by quantitative real-time (qRT)-PCR PCR. Real-time qRT-PCR of a series of differentiation-associated genes or metabolism related genes. Error bars are standard deviations. N = 3. **(B) **Venn diagram showing overlap among differentially expressed genes in Myc KD or Miz-1 KD ES cells. Gene ontology (GO) analysis of overlapping genes presented below the Venn diagram. *P *values are indicated.

To further study the effect of Myc loss-of-function on gene expression, we performed transcriptome analysis on day 5 Myc knockdown and control H9 human ES cells. Genes whose expression changed by at least 1.5-fold and that had a *P *value < 0.05 were considered differentially expressed. We found that 1,825 genes were upregulated and 1,060 genes were downregulated in c-Myc and/or N-Myc KD human ES cells [see Table S4 in Additional file 1]. Approximately 17% of the genes with changed expression upon Myc KD are Myc direct targets identified by our ChIP-chip studies. GO analysis indicated that genes involved in ES cell differentiation, Wnt signaling and apoptosis were upregulated, whereas genes important for transcriptional repression, signaling and negative regulation of differentiation were downregulated (Figure 5C, D). Of particular note, a large number of *Hox *genes (36 genes) were upregulated at least 1.5 fold upon disruption of Myc expression, with many being common genomic binding targets for both Myc and Miz-1 proteins. Importantly, Myc knockdown led to an upregulation in expression of approximately 10% of all Miz-1 direct target genes identified by ChIP-chip analysis (76 out of 735 genes) with the majority of them being involved in ES cell differentiation. We validated our results obtained by microarray analysis through quantitative real-time qRT-PCR (Figure 6A, B).

To study Myc and Miz-1 occupancy of *Hox *genes upon ES cell differentiation, we differentiated human ES cells into embryoid bodies (EBs). The ChIP analysis of Myc and Miz-1 binding to *Hox *genes in ES cells and EBs demonstrated a drastic reduction in Myc binding to all *Hox *genes tested in EBs, whereas Miz-1 binding remained mostly unchanged or only modestly decreased [see Figure S2A in Additional file 3]. Differentiation-associated gene expression was significantly upregulated in EBs compare to ES cells, as assessed by qRT-PCR analysis [see Figure S2B, C in Additional file 3].

### Evidence of transcriptional antagonism between Miz-1 and Myc

To measure the effects of Miz-1 KD on gene expression in human ES cells, we introduced shRNA specific to human Miz-1. We obtained a significant Miz-1 KD, which was confirmed by Western blot analysis [see Figure S3A in Additional file 4]. Miz-1 KD was not associated with any clear phenotypic changes in ES cell colony morphology [see Figure S3B in Additional file 4]. Gene expression array analysis on Miz-1 KD human ES cells revealed upregulation of 1,232 genes, with 11% of these genes being direct targets for Miz-1 as identified by ChIP-chip [see Table S5 in Additional file 1]. These upregulated genes comprised genes regulating mitochondrion biology, RNA metabolism, and cell cycle. Only 151 genes were downregulated upon Miz-1 knockdown, but importantly 16% of these genes were directly bound by Miz-1 and included *Hox *genes such as *MEIS1, HOXB5 *and *HOXB8*, and some apoptosis regulators.

We compared the gene expression changes in Miz-1 KD cells to those in Myc KD ES cells (Figure 6C). We found that 44 genes that are downregulated in Miz-1 KD human ES cells (29% of all genes downregulated in Miz-1 KD) are also upregulated with Myc KD in ES cells (*P *value = 0), suggesting a strong functional antagonism between Myc and Miz-1. The GO analysis revealed enrichment among these antagonistically regulated genes in *Hox *and developmental genes as well as in regulators of apoptosis. These results suggest that whereas the Myc/Miz-1 complex is likely to be responsible for the repression of developmental genes, Miz-1 is needed for activation of those genes. The lack of differentiation-associated phenotypic changes in Miz-1 KD human ES cells thus is likely due to the absence of Miz-1 that is required for the initiation of the differentiation program. The overlap between those genes independently upregulated in both the Myc KD and Miz-1 KD ES cells was not statistically significant (*P *value = 0.54). Interestingly, there are 57 genes that are upregulated in Miz-1 KD and downregulated in Myc KD human ES cells (*P *value = 0.0017), suggesting that in some contexts that Miz-1 may interfere with Myc transactivation, again supporting the notion of Myc and Miz-1 as antagonists. Importantly, only two genes were downregulated in both Myc KD and Miz-1 KD cells (*P *value = 0.22), suggesting that Myc and Miz-1 do not synergistically activate transcription of target genes.

### Myc interacts with Miz-1 and recruits Dnmt3a and HDACs

What is the mechanism by which Myc and Miz-1 act on transcription? The Myc/Miz-1 complex recruits Dnmt3a and HDACs to some target genes in immortalized cell lines [10,11]. To test whether Myc interacts with Miz-1, Dnmt3a and HDACs in human ES cells, we carried out coimmunoprecipitation (CoIP) with anti-c-Myc antibody followed by Western blot analysis of the endogenous Myc complexes in human ES cells. Our results indicated that endogenous c-Myc coprecipitates with Miz-1, HDAC1, HDAC2, and possibly HDAC3, as well as Dnmt3a [see Figure S4A in Additional file 5]. Myc did not interact with endogenous TRIM28, which was used as a negative control. These results indicate that Myc associates with several components of repression complexes in human ES cells. In addition to CoIP studies, we carried out ChIP assays using anti-HDAC antibodies, which revealed enrichment of HDACs on a significant subset of Miz-1/Myc target genes [see Figure S4B in Additional file 5].

## Discussion

Our functional genomics studies demonstrate that the coordinate functions of Myc and Miz-1 go well beyond the regulation of the limited set of genes previously identified by a candidate target gene approach. Myc/Miz-1 function involves a much broader set of targets involved in the maintenance of ES cell biology and more specifically in ES cell differentiation. Our findings also implicate *Hox *genes as key Myc target genes. *Hox *genes are repressed by Polycomb group proteins, which catalyze methylation at histone H3 lysine 27 (H3K27me3) of *Hox *genes to promote their repression in human ES cells [36]. Differentiation of ES cells leads to erasure of H3K27me3 marks and upregulation of *Hox *gene expression [37]. In our study we found that *Hox *gene expression is also repressed by the Myc/Miz-1 complex, which represents a novel mechanism of *Hox *gene repression in stem cells. The *Hox *genes targeted by Myc and Miz-1 possessed enrichment for H3K27me3 marks as well, suggesting a potential link between Myc and Polycomb as has been observed in *Drosophila *[38].

Recent studies in mouse ES cells defined three functionally separate ES modules: Core (74 genes), Polycomb (449 genes), and Myc (355 genes) [39] suggesting that Myc largely functions independently of Polycomb. We found that out of 4,398 Myc target genes identified in our study in human ES cells, 862 genes (20%) are also identified as Myc target genes in the study by Kim *et al. *in mouse ES cells. We have identified 119 Myc target genes that fell into the Polycomb module, 84 target genes that fell into Myc module and 13 target genes belonging to the Core module. The enrichment of the genes identified as belonging to the Polycomb module in identified Myc target genes in our study further suggest the potential link between Myc and Polycomb in human ES cells. An additional study [40] found that Myc exhibits substantial overlap in genomic binding in mouse ES cells with Polycomb binding, but Myc activated those Polycomb-targeted genes and had no effect on H3K27me3 levels. These findings and our data here leave open the precise role, if any, of Myc together with Polycomb in ES cells, but at least suggest that Myc and Polycomb are cooperative in ES cells, most likely not through regulating H3K27me3.

We found evidence of a complex relationship between Myc and cell survival in ES cells. The link between Myc and apoptosis is well documented [41]. While Myc overexpression is linked to cell death in some contexts, so is the loss of Myc in other contexts [42]. Our ChIP-chip study demonstrates that Myc directly controls the expression of a large number of cell death related genes. The absence of Myc, however, also leads to the activation of proapoptotic program, which can be attributed to the global dysregulation of a variety of cellular pathways that in turn elicits secondary response involving activation of a cell death program.

Our findings on localization of Miz-1 genomic binding almost exclusively away from TSS were particularly unexpected and of interest because the predominant model in the field is that Miz-1 exclusively targets INR sequences in the immediate proximity of TSS [1,43]. However, these previous studies only examined core promoter regions of a few known candidate Miz-1 target genes, likely not fully representing the global distribution of Miz-1 genomic binding and function.

What might be the mechanism underlying the Miz-1 INR-independent function? One possibility is that Miz-1 is recruited to the upstream promoter region through interaction with other DNA-binding proteins. Alternatively, Miz-1 may directly bind as yet unknown motifs that are distinct from INRs in terms of both location and sequence. We were not able to identify any novel predicted consensus motifs for Miz-1 binding within Myc and Miz-1 cobound regions by MEME algorithms, but this may reflect the expected large size and complexity of Miz-1 DNA binding sites as it has 13 zinc finger DNA binding domains. Since we did not find Myc E-boxes significantly enriched in Myc/Miz-1 cobound sequences, it remains unclear if Myc binds DNA directly in such complexes.

More generally, we found two main types of Miz-1 genomic targets in human ES cells: (1) targets of putative Miz-1 activation that are associated strongly with euchromatic, transcriptionally active histone marks, and (2) targets of possible Miz-1 repression that lack or have very low relative levels of euchromatic marks. The pool of former, active genomic domains bound by Miz-1 comprises genes involved in regulation of metabolism, cell cycle control, chromatin and protein ubiquitination, indicating potential mechanisms by which Miz-1 regulates ES cell biology in a Myc-independent manner. A relatively small group of Miz-1 target genes (36 genes out of 734 genes) displayed simultaneous enrichment in activating and silencing histone modifications, including several developmentally regulated genes such as *MEIS2, PAX6, GSC *and *SHOX2*. These genes might be poised, having 'bivalent domains', which harbor both activation and silencing marks [44]. Myc binding to a subset of genes within 'bivalent domains' was previously reported [45,46], but here we implicate Miz-1 in this process.

Another model is that Myc and Miz-1 are strictly antagonistic. Consistent with this model, Myc loss-of-function in human ES cells leads to increased expression of many Myc/Miz-1 targets. Interestingly, Myc and Miz-1 may also be antagonistic in another, novel way in the context of Myc activation. We found a statistically very significant group of Myc targets of activation that were not only downregulated by loss of Myc, but also upregulated by loss of Miz-1. If Miz-1 indeed functions to antagonize transactivation by Myc of a specific subset of target genes, it must be through an as yet new mechanism of action by Miz-1 as it has not been previously reported to repress transcription or recruit corepressors. The lack of a statistically significant population of genes that are either downregulated in both Myc and Miz-1 KD, or upregulated in both KDs, clearly demonstrates that Myc and Miz-1 do not act synergistically to either activate or repress the expression of their target genes. This further supports the model of Myc and Miz-1 generally being antagonists.

## Conclusions

**Figure 7:**
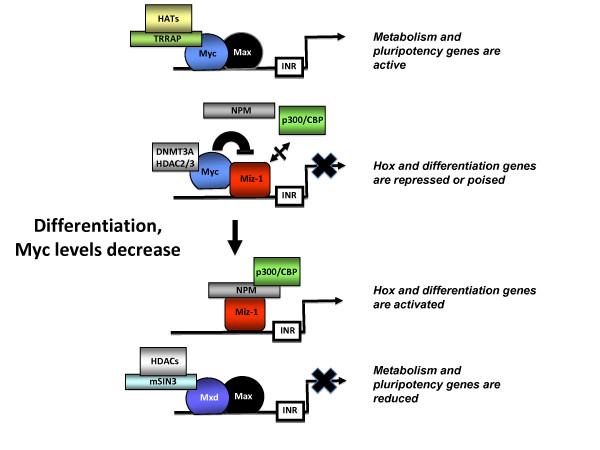
**Model**. Myc/Miz-1 pathway acts as a master switch that represses multiple differentiation-associated genes.

In the present work, we have provided important insight into the mechanisms by which Myc represses differentiation genes to promote pluripotency: a previously unknown role of the Myc/Miz-1 complex in repression of differentiation-related genes in human ES cells. We propose a model whereby the Myc/Miz-1 pathway acts as a master switch that represses multiple differentiation related genes (Figure 7). In this model we hypothesize that Myc expression counteracts Miz-1 transactivation of *Hox *and other differentiation-promoting genes thus maintaining ES cell pluripotency. In the presence of prodifferentiation signals, Myc levels are decreased, which leads to activation of Miz-1 bound differentiation genes and results in initiation of coordinated differentiation program. A clearer understanding of the potential roles of the Myc/Miz-1 complex in tumorigenesis and iPS cell formation awaits future studies including functional genomics analyses of endogenous Miz-1 and Myc in other cell types.

## Methods

### Cell culture

The human ES cell line H9 was maintained in ES medium on irradiated mouse embryonic fibroblasts (Global Stem, Rockville, MD). Cells were passaged by treatment with 1 mg/ml collagenase (Gibco, Carlsbad, CA).

### Chromatin immunoprecipitation and ChIP-chip

ChIPs were conducted on H9 human ES cells as previously described [47]. Then, 3 μg of one of the following antibodies was added to each diluted fraction: anti-N-Myc (Santa Cruz sc53993, Santa Cruz, CA), anti-c-Myc (Abcam ab17355, San Francisco, CA), anti-AcH3K9 (Upstate 06-942, Billerica, MA), anti-H3K4me3 (Upstate 04-745, Billerica, MA), anti-HDAC 1 (Abcam ab31263, San Francisco, CA), anti-HDAC 2 (Abcam ab12169, San Francisco, CA), anti-HDAC 3 (Abcam ab7030, San Francisco, CA), anti-Miz-1 (Santa Cruz, sc-22837, Santa Cruz, CA) or rabbit IgG or mouse IgG. We validated that N-Myc and c-Myc antibodies were not crossreactive. For Myc and Miz-1 ChIP-chip experiments, peaks with the score ≥ 1 and FDR ≤ 0.05 were considered positive targets for protein binding. For AcH3K9 and H3K4me4 ChIP-chip experiments, peaks with the score ≥ 2, FDR ≤ 0.05 were considered positive targets for protein binding.

### Coimmunoprecipitation and western blot analysis

Cells were lysed in lysis buffer (50 mM 4-(2-hydroxyethyl)-1-piperazineethanesulfonic acid (Hepes) pH 7.9, 1 mM MgCl_2_, 20 mM NaCl, 10 mM ethylenediaminetetra-acetic acid (EDTA) pH 8.0, 10% glycerol, 0.2% nonyl phenoxypolyethoxylethanol (NP-40), 1 × protease inhibitor cocktail). Whole-cell lysates were subjected to immunoprecipitation using the anti-c-Myc antibody (Abcam, San Francisco, CA) overnight. Immunocomplexes were recovered with protein-G-sepharose beads (GE Healthcare, Piscataway, NJ). Samples were resolved by SDS-PAGE before transfer to polyvinylidene fluoride (PVDF) membrane. The membranes were incubated with the appropriate antibodies.

### RNA interference

293T cells were transfected with the pLKO.1 lentiviral constructs containing the shRNAs against human c-Myc and N-Myc (Sigma Aldrich, St. Luis, MO) along with the packaging plasmids (pMD.G and Delta 8.9), using FuGene HD (Roche, Indianapolis, IN). Empty pLKO.1 construct was used as a control. For Miz-1 knockdown experiments, platA packaging cells (Cell Biolabs, Inc, San Diego, CA) were transduced with pRFP-C-RS vectors containing either scrambled shRNA or shRNA specific to Miz-1. Supernatants containing the viruses were harvested 48 h and 72 h after transfection. Human ES cells H9 were infected with the viral supernatants in the presence of 6 μg/ml of polybrene and selected with 1 μg/ml puromycin 48 h later.

### qPCR and gene expression array analysis

Total RNA was isolated from human ES cells using the RNeasy Plus mini kit (Qiagen, Valencia, CA) as described by manufacturer. RNA was reverse transcribed by using Superscript III First strand synthesis Supermix (Invitrogen, Carlsbad, CA). qPCR assays were performed in triplicate using Absolute Blue qPCR SYBR Green Mix (Fisher Scientific, Pittsburgh, PA), except for Myc assays, which were performed using TaqMan gene expression assays (Applied Biosystems, Foster City, CA) on a Roche LightCycler 480 (Indianapolis, IN). Expression was normalized using peptidylprolyl isomerase A (PPIA; cyclophilin A) endogenous control. Sample data was analyzed using comparative Ct method and standard deviation calculated based on Applied Biosystems methods (Bulletin 04371095).

For gene expression analysis, isolated RNA (40 ng/μl) from Myc KD, Miz-1 KD and control ES cells was submitted to the UC Davis Expression Analysis Core for gene expression analysis using Illumina Centrix Human-6 Beadchip, San Diego, CA. Average normalization was applied to the expression data from the three biological replicates. Genes whose expression changed by at least 1.5-fold and a *P *value < 0.05 were considered differentially expressed. For Miz-1 knockdown experiments, genes whose expression changed by at least 1.3-fold and a *P *value < 0.05 were considered differentially expressed. Obtained results were analyzed with DAVID V6.7 Functional Annotation Bioinformatics Microarray software.

### *In vitro *differentiation to embryoid bodies (EBs)

Colonies of human ES cells were dislodged from plates by treatment with 1 mg/ml of collagenase (Gibco, Carlsbad, CA). Cells were washed and resuspended in differentiation medium (Dulbecco's modified Eagle medium (DMEM) High Glucose (Invitrogen, Carlsbad, CA), 20% fetal bovine serum (FBS) (Hyclone, Lakewood, NJ), 100 μM minimal essential medium (MEM) non-essential amino acids (Invitrogen, Carlsbad, CA), 2 mM glutamine (Invitrogen, Carlsbad, CA), and 100 μM β-mercaptoethanol (Invitrogen, Carlsbad, CA). Resuspended cells were plated in low-attachment 10 cm dishes. EBs were collected for analysis 10 days later.

## Competing interests

The authors declare that they have no competing interests.

## Authors' contributions

PK and NV conceived the studies and the experimental design. NV conducted most of the experiments with RC performing the ChIP-chip work. KB and IK conducted some of the statistical analysis. NV and PK performed the data analysis and wrote the manuscript. All authors read and approved the manuscript.

## Supplementary Material

Additional file 1 **Tables S1 to S5**. Table S1. Miz-1 direct targets. Table S2. Myc direct targets. Table S3. Miz-1 and Myc targets. Table S4. Myc knockdown gene expression changes. Table S5. Miz-1 knockdown gene expression changes. Click here for file

Additional file 2 **Figure S1**. Confirmation of Myc knockdown in human embryonic stem (ES) cells by western blot analysis. Click here for file

Additional file 3 **Figure S2**. Differentiation of human embryonic stem (ES) cells into embryoid bodies (EBs) leads to a drastic reduction of levels of Myc bound to *Hox *genes, and a significant upregulation of differentiation-associated genes. **(A) **Chromatin immunoprecipitation (ChIP) analysis of N-Myc and Miz-1 binding in human ES cells and EBs. **(B, C) **Real-time quantitative real-time (qRT)-PCR PCR of a series of differentiation-associated genes. Click here for file

Additional file 4 **Figure S3**. Confirmation of Miz-1 knockdown (KD) in human embryonic stem (ES) cells. **(A) **Western blot analysis of Miz-1 protein levels. **(B) **Phase contrast images of human ES cells transduced with either scrambled small hairpin (sh)RNA control or shRNA specific to Miz-1. Images were taken at 4 × magnification. Click here for file

Additional file 5 **Figure S4**. Myc associates with Miz-1, HDACs, and DNMT3A *in vivo *in human embryonic stem (ES) cells. **(A) **Coimmunoprecipitation with a c-myc antibody or a non-specific IgG. Western blot analysis was performed using antibodies specific to Miz-1, Dnmt3a, HDAC1, HDAC2, and HDAC3. TRIM28 was used as a negative control for the interaction with Myc. (B) Chromatin immunoprecipitation (ChIP) on Myc and Miz-1 cobound gene targets. Click here for file
