# Supplementary figures and images for: Myc and Miz-1 have coordinate genomic functions including targeting Hox genes in human embryonic stem cells
Source: Epigenetics Chromatin. 2011 Nov 4;4:20. doi: 10.1186/1756-8935-4-20 (PMC3226433; doi:10.1186/1756-8935-4-20)

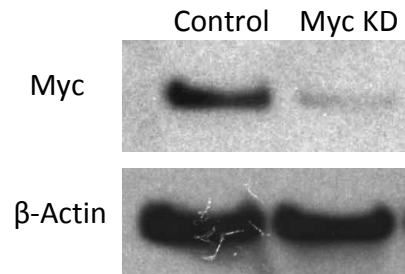

Figure S1. Confirmation of Myc knockdown in human ES cells by Western Blot analysis.

Supplement: Additional file 2 — Figure S1. Confirmation of Myc knockdown in human embryonic stem (ES) cells by western blot analysis. [file 1756-8935-4-20-S2.PDF]
