# Supplementary material for: Myc and Miz-1 have coordinate genomic functions including targeting Hox genes in human embryonic stem cells
Source: Epigenetics Chromatin. 2011 Nov 4;4:20. doi: 10.1186/1756-8935-4-20 (PMC3226433; doi:10.1186/1756-8935-4-20)
Supplement: Additional file 3 — Figure S2. Differentiation of human embryonic stem (ES) cells into embryoid bodies (EBs) leads to a drastic reduction of levels of Myc bound to Hox genes, and a significant upregulation of differentiation-associated genes. (A) Chromatin immunoprecipitation (ChIP) analysis of N-Myc and Miz-1 binding in human ES cells and EBs. (B, C) Real-time quantitative real-time (qRT)-PCR PCR of a series of differentiation-associated genes. [file 1756-8935-4-20-S3.PDF]

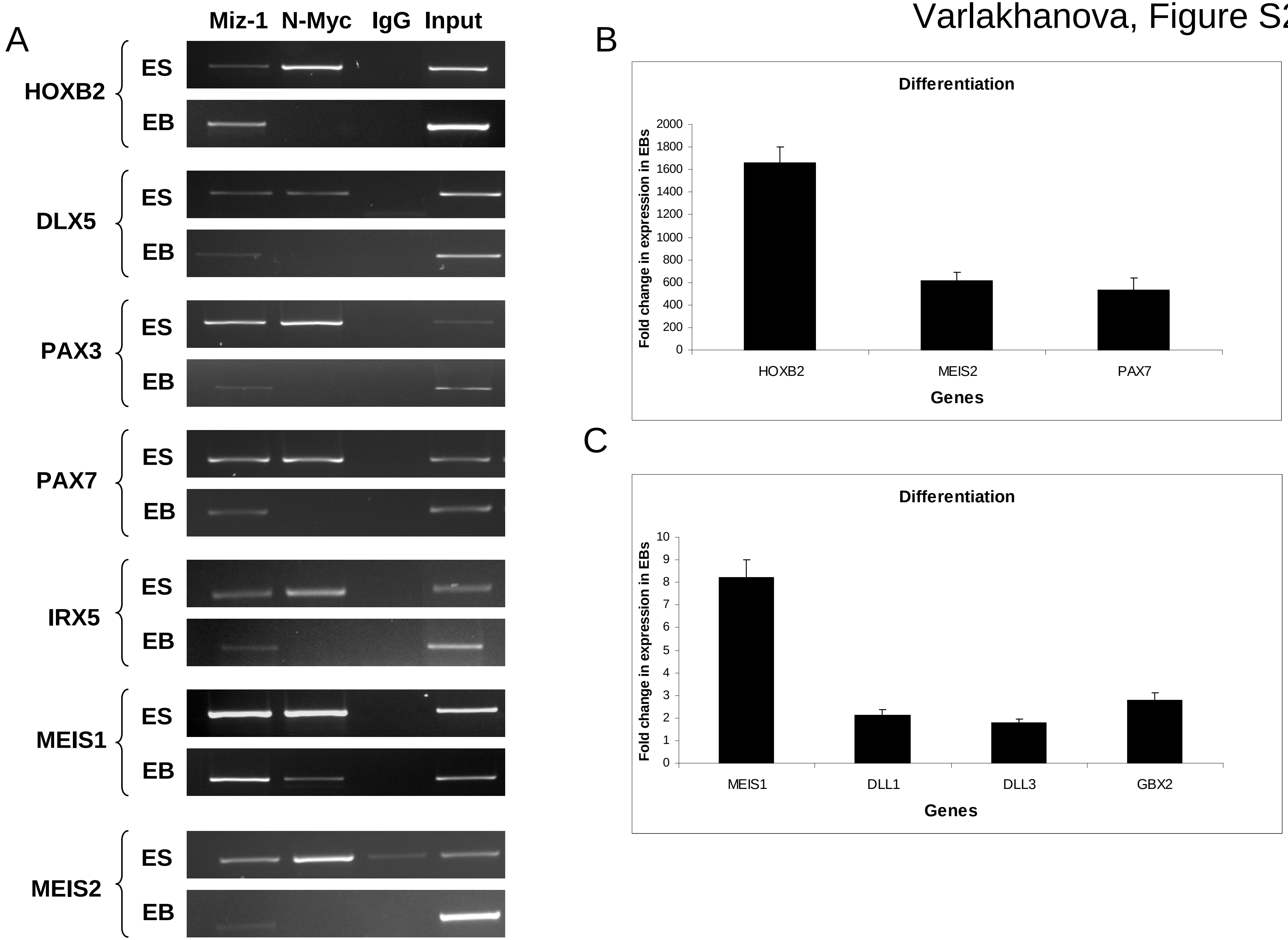

Figure S2. Human Embryoid Bodies (EBs) exhibit loss of Myc binding on *Hox* genes, and a significant up-regulation of differentiation-associated genes. A. ChIP analysis of N-Myc and Miz-1 binding in human ES cells and EBs. B and C. Real-time qRT-PCR of a series of differentiation associated genes. Error bars are standard deviations. N=3.
