# Supplementary material for: Myc and Miz-1 have coordinate genomic functions including targeting Hox genes in human embryonic stem cells
Source: Epigenetics Chromatin. 2011 Nov 4;4:20. doi: 10.1186/1756-8935-4-20 (PMC3226433; doi:10.1186/1756-8935-4-20)
Supplement: Additional file 4 — Figure S3. Confirmation of Miz-1 knockdown (KD) in human embryonic stem (ES) cells. (A) Western blot analysis of Miz-1 protein levels. (B) Phase contrast images of human ES cells transduced with either scrambled small hairpin (sh)RNA control or shRNA specific to Miz-1. Images were taken at 4 × magnification. [file 1756-8935-4-20-S4.PDF]

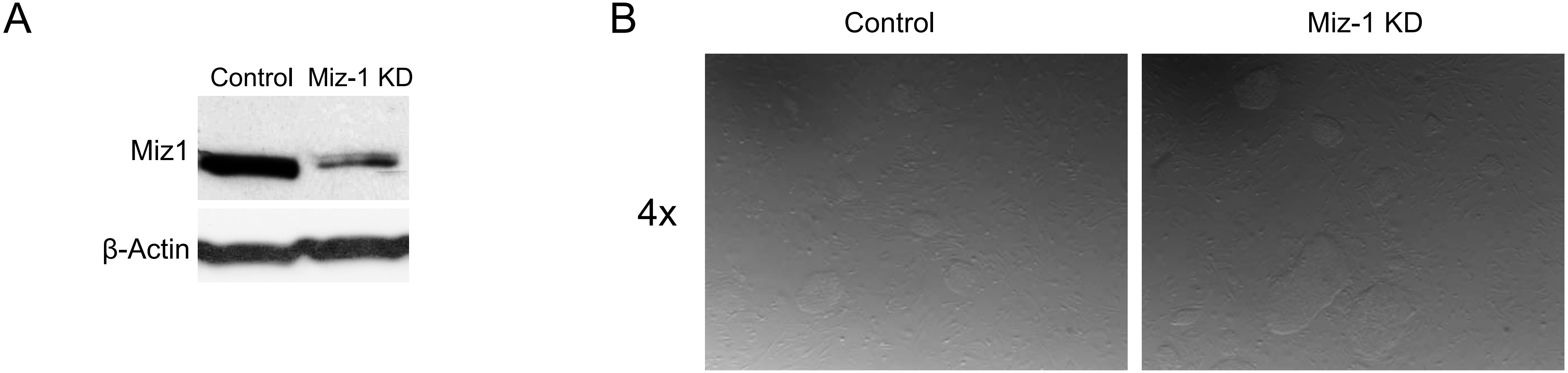

Figure S3. Confirmation of Miz-1 KD in human ES cell. A. Western blot analysis of Miz-1 protein levels. B. Phase contrast images of human ES cells transduced with either scrambled shRNA control or shRNA specific to Miz-1. Images were taken at 4x magnification.
