# Supplementary material for: Myc and Miz-1 have coordinate genomic functions including targeting Hox genes in human embryonic stem cells
Source: Epigenetics Chromatin. 2011 Nov 4;4:20. doi: 10.1186/1756-8935-4-20 (PMC3226433; doi:10.1186/1756-8935-4-20)
Supplement: Additional file 5 — Figure S4. Myc associates with Miz-1, HDACs, and DNMT3A in vivo in human embryonic stem (ES) cells. (A) Coimmunoprecipitation with a c-myc antibody or a non-specific IgG. Western blot analysis was performed using antibodies specific to Miz-1, Dnmt3a, HDAC1, HDAC2, and HDAC3. TRIM28 was used as a negative control for the interaction with Myc. (B) Chromatin immunoprecipitation (ChIP) on Myc and Miz-1 cobound gene targets. [file 1756-8935-4-20-S5.PDF]

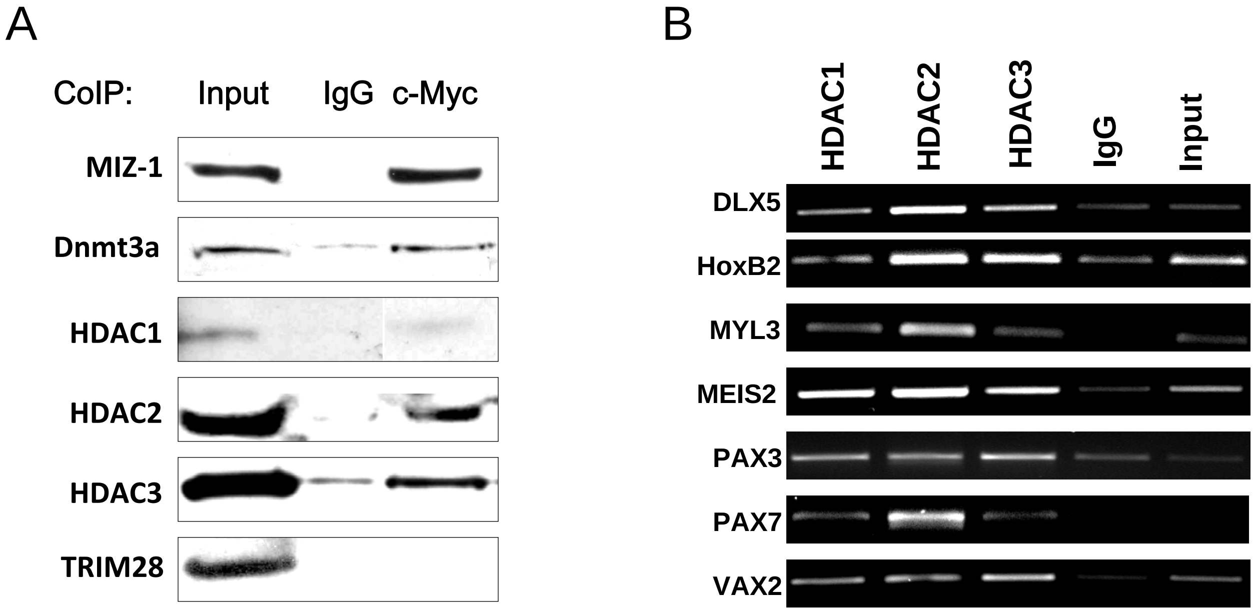


**Figure S4. Myc associates with Miz-1, HDACs, and DNMT3A in vivo in human ES cells.** A. Co-immunoprecipitation with a c-myc antibody or a non-specific IgG. Western blot analysis was performed using antibodies specific to Miz-1, Dnmt3a, HDAC1, HDAC2, HDAC3. TRIM28 was used as a negative control for the interaction with Myc. B. ChIP on Myc and Miz-1 co-bound gene targets.
